# How Do Health and Social Networks Compare between Low-Income Multiproblem Households and the General Population?

**DOI:** 10.3390/ijerph16244967

**Published:** 2019-12-06

**Authors:** Gera E. Nagelhout, Latifa Abidi, Hein de Vries

**Affiliations:** 1 Department of Health Promotion, Maastricht University (CAPHRI), 6200 Maastricht, Limburg, The Netherlands; latifa.abidi@maastrichtuniversity.nl (L.A.); hein.devries@maastrichtuniversity.nl (H.d.V.); 2 Department of Family Medicine, Maastricht University (CAPHRI), 6200 Maastricht, Limburg, The Netherlands; 3 IVO Research Institute, 2490 The Hague, Zuid–Holland, The Netherlands

**Keywords:** Netherlands, multiproblem households, health behavior, mental health, social network, social class

## Abstract

Multiproblem households that receive social care for multiple problems, such as debts, psychiatric disorders, and domestic violence, may also be disadvantaged in terms of health and social networks. This study examines whether low-income multiproblem households and the general population differ in self-perceived health, mental health, health behaviors, and social networks. We performed a cross-sectional survey among respondents from low-income multiproblem households (n = 105) and the general population (n = 99) in the municipality of Apeldoorn in the Netherlands. Comparisons with national statistics data indicated that our sample of multiproblem households is more disadvantaged in terms of self-perceived health and mental health than low socioeconomic groups in general in the Netherlands. A multiple logistic regression analysis showed that being part of the multiproblem household group versus the general population group was associated with a lower educational level, a lower likelihood of being in paid employment, a lower score with respect to mental health, less alcohol consumption, and less fruit consumption. There were also differences between the groups on other variables, but these were not significant in adjusted analyses. In conclusion, multiproblem households in Apeldoorn had lower scores on mental health, drank fewer alcoholic drinks per week, and ate less fruit than the general population.

## 1. Introduction

Multiproblem households that receive social care for multiple problems in their family lives, such as debts, psychiatric disorders, and domestic violence, may also be disadvantaged in terms of health and social networks. Gaining insight into health and social network indicators of this vulnerable group can inform preventive activities, such as community-based health promotion programs.

Research among multiproblem households has been limited to a few studies from the United States that only examined substance use behaviors and the mental health of this group [1,2,3] but no other indicators of healthy living, such as physical activity, nutrition, and self-perceived general health. One study from Portugal examined social networks among multiproblem households [4] but did not compare this with the general population. One study from the Netherlands compared mental health care and social network problems between multiproblem households referred to youth mental health care and the general population [5] but did not examine other indicators. There is a need for studies that examine a broader range of health and social network indicators among multiproblem households in comparison with the general population to inform the development of health promotion programs for this group.

This study compares multiproblem households and the general population living in one municipality in the Netherlands. As described above, most other studies have been performed in the United States (USA), where healthcare is less accessible and of lower quality than in the Netherlands. Multiproblem households in the Netherlands receive intensive family home care support from specialized social workers. It is unknown whether multiproblem households living in such a healthcare system differ from the general population in self-perceived health, mental health, health behaviors, and social networks.

## 2. Materials and Methods

### 2.1. Sample

Data were collected from two samples. The first sample consisted of low-income multiproblem household members from Apeldoorn in the Netherlands. The second sample consisted of the general population from Apeldoorn in the Netherlands. The first sample was part of a quasi-experimental intervention study [6], but in the current study we only used data from the baseline questionnaire. The study protocol of this study was approved by the Medical Ethics Committee of Zuyderland and Zuyd Hogeschool (METC number: 17–N–80).

Low-income multiproblem household members (n = 105) were recruited between September 2017 and July 2018 by their social workers, by staff of a local welfare organization, or by a researcher. Inclusion criteria were a disposable income up to 150% of the minimum wage, receipt of social care for problems in more than one area, residence in Apeldoorn, and an age of 16 years or older [6]. Everyone from the household who was aged 16 years or older could participate in the study, but typically only one or two persons from the household did (people from 93 households participated). The paper-and-pencil questionnaires were administered by the researchers. The respondents from this group received a filled grocery bag with a value of €15 for their participation in the study. Additionally, all minors who participated received a coupon of €10 that they could spend in a budget sports store.

Members of the general population (n = 99) were recruited between October 2017 and March 2018 by two students and were approached on several streets in Apeldoorn. The inclusion criteria were residence in Apeldoorn and an age of 16 years or older (this was asked before filling in the questionnaire). An exclusion criterion was the reporting of social care for problems in more than one area (this was assessed in the questionnaire). The paper-and-pencil questionnaires were self-administered. One coupon of €50 was raffled among the respondents from this group.

The two samples from Apeldoorn were not only compared with each other but also with national statistics data from the Netherlands. These were collected through the internet by Statistics Netherlands in 2018 (CBS). A random sample of about 9500 inhabitants aged 12 years and older per year was drawn using the national registry as a sampling frame. Initial nonresponders were approached to complete the survey face to face.

### 2.2. Measurements

The sociodemographic characteristics we measured were gender (woman or man), age, educational level, and paid employment (yes or no). Educational level was categorized as low (primary education or lower prevocational secondary education), moderate (middle prevocational and secondary vocational education), and high (senior general secondary education, higher professional education, and university).

Self-perceived health was measured with one question: “How is your health condition generally?” (with a response scale from 1 ‘very bad’ to 5 ‘very good’) [7]. Smiley faces were added above the scale to facilitate interpretation. Mental health was measured using the five-item Mental Health Inventory [8]. Tobacco consumption was measured by asking about daily smoking (yes or no). Alcohol consumption was measured using the Dutch Quantity-Frequency-Variability (QFV) Questionnaire [9] and resulted in the number of alcoholic drinks consumed per week. Physical activity was measured using the International Physical Activity Questionnaire (IPAQ) questionnaire [10] and was dichotomized into 2.5 h per week or more (i.e., enough physical activity according to the Dutch activity guidelines) versus less than 2.5 h per week. Vegetable and fruit consumption was measured with questions from the Dutch Public Health Monitor and resulted in the number of serving spoons of vegetables and number of pieces of fruit per week. Body mass index (BMI) was calculated after asking the respondents for their height and weight. Social contacts were measured with a three-item index from the Dutch Public Health Monitor [11], and loneliness was measured with a six-item scale for overall, emotional, and social loneliness [12]. Cronbach’s alpha for the loneliness scale was 0.88.

### 2.3. Analyses

Chi-square tests were performed to compare the two study samples with national statistics data for self-perceived health and mental health. For these analyses, self-perceived health was dichotomized into good and very good (1) versus the rest (0), and mental health was dichotomized into lower than 60 (0) and 60 or higher (1). Additionally, Chi-square tests and independent sample t-tests were performed to compare the two study samples on all sociodemographic characteristics and health indicators. Additionally, a multiple logistic regression analysis was performed with each group (multiproblem versus general population) as a dependent variable and all other variables as independent variables.

## 3. Results

### 3.1. Comparison with National Statistics Data

**Figure 1:**
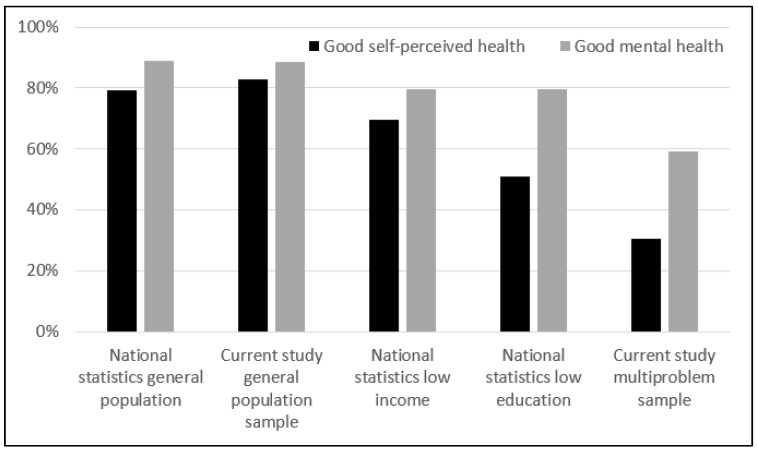
Differences in self-perceived health and mental health between the following groups: national statistics general population, the current study general population sample, national statistics low-income, national statistics low education, and the current study multiproblem sample.

The percentage of people with good self-perceived health (χ^2^ = 0.40, *p* = 0.528) and good mental health (χ^2^ = 0.01, *p* = 0.944) were comparable between national statistics data from the general population and the current study sample of the general population (Figure 1). The respondents from multiproblem households of the current study sample had worse self-perceived health than national statistics data from low-income (χ^2^ = 29.33, *p* < 0.001) and low education groups (χ^2^ = 7.88, *p* = 0.005). The respondents from multiproblem households of the current study sample had worse mental health than national statistics data from low-income (χ^2^ = 9.03, *p* = 0.003) and low education groups (χ^2^ = 9.03, *p* = 0.003).

### 3.2. Comparison between the Two Study Samples

Respondents from multiproblem households were on average older, had a lower educational level, were more often unemployed, had worse self-perceived health and mental health, were more often daily smokers, drank fewer alcoholic drinks per week, were less physically active, ate fewer vegetables and fruit, had a higher BMI, had fewer social contacts, and perceived more loneliness than the respondents from the general population (Table 1).

Multiple logistic regression analysis showed that being part of the multiproblem household group versus the general population (reference) group was associated with a lower educational level (OR = 15.62, *p* = 0.005 for low and OR = 4.81, *p* = 0.048 for moderate compared with higher educated), a lower likelihood of being in paid employment (OR = 0.03, *p* < 0.001), a lower score with respect to mental health (OR = 0.96, *p* = 0.031), fewer alcoholic drinks per week (OR = 0.93, *p* = 0.048), and less fruit consumption (OR = 0.85, *p* = 0.004).

## 4. Discussion

Our study compared people living in low-income households who received social care for multiple problems with the general population in terms of self-perceived health, mental health, health behaviors, and social networks. Our results revealed that people from multiproblem households scored lower with respect to mental health (62 versus 77 on a scale from 0 to 100), drank fewer alcoholic drinks per week (on average three versus seven), and ate fewer fruits per week (on average six versus nine) than the general population. These results are in line with comparisons in health and health behaviors between high and low socioeconomic groups, e.g., [13,14].

We found fewer social contacts and more loneliness among people from multiproblem households than among the general population, but these differences were no longer significant after adjusting for other characteristics, such as educational level and employment status. A Portuguese study about social networks among multiproblem households suggested that multiproblem households have relatively unstable social networks and are prone to social isolation among other issues due to their low employment levels [4]. Focusing on mental health and social networks is already a part of the social care of multiproblem households but not always a part of health promotion programs. It is important that future health promotion programs for this group take this into account. Additionally, people living in low-income households who received social care for multiple problems tend to be lower educated and less often have paid employment than the general population. The lives of people living in multiproblem households can potentially be improved by helping them with education and job opportunities. This is already part of the social care that is received. However, on a municipality or national policy level, education and job opportunities could be created for people in a more vulnerable position.

In a previous study among the same group, we found that alcohol was not often used because of difficult past experiences, such as violence and abuse by people with problematic alcohol use in their social environment, particularly among women [15]. Our results with respect to alcohol consumption among multiproblem households from this and the previous study may have been influenced by selection bias; people from multiproblem households with problematic alcohol use may not have wanted to participate in our studies. Therefore, we cannot conclude for certain whether health promotion programs for multiproblem households should focus on alcohol use or not.

National statistics data were available for self-perceived health and mental health for the general population from the Netherlands as well as for the low-income and low-educated Dutch population. Comparisons with our two study samples indicated that our general population sample from the municipality Apeldoorn was representative of the general population in the Netherlands in terms of self-perceived health and mental health. Additionally, it showed that the group of multiproblem households was more disadvantaged in terms of self-perceived health and mental health than low socioeconomic groups in general in the Netherlands. This can be explained by the fact that members of multiproblem households not only have socioeconomic stressors in their lives but also stressors such as domestic violence, delinquent behavior of family members, problems related to childcare, and traumas due to a troubled youth [13]. In a previous study, we have shown that these stressors are considered barriers for health behavior change among adults from multiproblem households [15].

A strength of our study is that we examined a range of health and social network indicators among multiproblem households and compared this with the general population. However, our study also has limitations. It was not feasible to perform random sampling, and therefore, we cannot be sure about the generalizability of our findings. All concepts in our study were self-reported, and the presence of an interviewer may have made people more prone to providing socially desirable responses. Our sample of people from multiproblem households was part of an intervention study that only included people with a low disposable income, making it not possible to fully disentangle socioeconomic differences from differences between groups with multiple problems and without multiple problems. The intervention study was to evaluate a health promotion program. Although the respondents themselves were not aware of this before they filled in the baseline questionnaire that we used for the current analysis, it is possible that their social workers selected them for this study because of health problems. This could have affected our results. Additionally, there is an element of circularity in our questions, examining whether people from multiproblem households have mental health problems, while one of the multiple problems that they have may indeed be mental health problems. Finally, both samples were relatively small.

## 5. Conclusions

Based on the results from our study, it can be concluded that health promotion programs for low-income multiproblem households should especially focus on improving mental health and increasing fruit consumption among this target group. It does not seem that alcohol use is more problematic among this group than among the general population in the Netherlands. Health promotors could also consider focusing on improving self-perceived health, stimulating smoking cessation, encouraging physical activity, promoting vegetable consumption, and improving social networks of multiproblem households. There were differences in these health indicators between low-income multiproblem households and the general population, although these may primarily be caused by the lower socioeconomic status of the first group.

## Figures and Tables

**Table 1:** Differences in sociodemographic characteristics and health indicators among the respondents from multiproblem households and the respondents from the general population.

Variable	Multiproblem Households (n = 105)	General Population (n = 99)	Unadjusted Analyses	Adjusted Analysis
**Sociodemographic characteristics**				
Gender				
Women (%)	59.0	56.6	χ^2^ = 0.13, *p* = 0.720	OR = 2.16, *p* = 0.251
Men (%)	41.0	43.4		Ref.
Age				
Mean (SD)	44.9 (13.1)	38.2 (15.2)	*t* = −3.36, *p* < 0.001	OR = 1.02, *p* = 0.425
Educational level				
Low (%)	44.2	12.1	χ^2^ = 39.13, *p* < 0.001	OR = 15.62, *p* = 0.005
Moderate (%)	41.3	37.4		OR = 4.81, *p* = 0.048
High (%)	14.4	50.5		Ref.
Paid employment				
Yes (%)	21.9	90.7	χ^2^ = 93.04, *p* < 0.001	OR = 0.03, *p* < 0.001
No (%)	78.1	9.3		Ref.
**Health indicators**				
Self-perceived health (1–5)				
Mean (SD)	3.1 (0.8)	4.0 (0.7)	*t* = 8.62, *p* < 0.001	OR = 0.65, *p* = 0.257
Mental health (0–100)				
Mean (SD)	61.6 (22.4)	76.8 (14.7)	*t* = 5.72, *p* < 0.001	OR = 0.96, *p* = 0.031
Daily smoking				
Yes (%)	42.9	24.7	χ^2^ = 7.36, *p* = 0.007	OR = 3.03, *p* = 0.127
No (%)	57.1	75.3		Ref.
Alcoholic drinks per week				
Mean (SD)	3.1 (8.2)	6.5 (9.3)	*t* = 2.74, *p* = 0.007	OR = 0.93, *p* = 0.048
Physical activity				
2.5 h per week or more (%)	81.0	94.8	χ^2^ = 8.97, *p* = 0.003	OR = 1.70, *p* = 0.587
Less than 2.5 h per week (%)	19.0	5.2		Ref.
Vegetable consumption (serving spoons per week)				
Mean (SD)	11.9 (7.5)	14.7 (7.1)	*t* = 2.72, *p* = 0.007	OR = 1.03, *p* = 0.524
Fruit consumption (pieces per week)				
Mean (SD)	6.0 (6.3)	9.3 (6.4)	*t* = 3.74, *p* < 0.001	OR = 0.85, *p* = 0.004
Body Mass Index (BMI)				
Mean (SD)	28.2 (6.5)	24.2 (3.2)	*t* = −5.57, *p* < 0.001	OR = 1.11, *p* = 0.178
Social contacts (1–5)				
Mean (SD)	4.6 (1.3)	5.3 (0.7)	*t* = 5.18, *p* < 0.001	OR = 0.65, *p* = 0.268
Loneliness (1–5)				
Mean (SD)	2.6 (0.9)	1.8 (0.7)	*t* = −7.42, *p* < 0.001	OR = 2.35, *p* = 0.062
